# High-Grade Malignant Spindle Cell Sarcoma of the Pelvis Presenting With Bloody Diarrhea and Urinary Retention: A Rare Case With a Rare Presentation

**DOI:** 10.7759/cureus.10622

**Published:** 2020-09-23

**Authors:** Ali Jawad Jang Khan, Noman Ahmed Jang Khan

**Affiliations:** 1 Medicine, Dow Medical College/Civil Hospital, Karachi, PAK; 2 Hematology and Oncology, Joan C. Edwards School of Medicine, Marshall University, Huntington, USA

**Keywords:** sarcoma soft tissue, spindle cell

## Abstract

Soft tissue sarcomas (STSs) are rare malignant tumors originating from mesenchymal cells. Extremities are the most commonly affected anatomical sites, and majority of them present as a painless mass. We present a very interesting case of high-grade spindle cell sarcoma of the pelvis manifested as urinary retention and bloody diarrhea. A 68-year-old male presented to the emergency department with abdominal pain, inability to void urine, and bloody diarrhea. Straight urinary catheterization retrieved 900 mL of urine, and a Foley catheter was placed. All laboratory workup including complete blood count, complete metabolic panel, and urinalysis were within normal limits, but computed tomography (CT) of the abdomen and pelvis with contrast was remarkable for bilateral moderate hydronephrosis and a large 14 x 9.1 cm pelvic mass fistulizing into the rectum. To better identify the extent of disease, magnetic resonance imaging (MRI) with contrast was performed, which also revealed a similar large pelvic mass fistulizing into the rectum. Core needle biopsy of the mass was performed, which showed malignant spindle and epithelioid neoplasm with necrosis consistent with high-grade sarcoma. This is a very rare presentation of STS, and, to the best of our knowledge, only few similar cases have been reported thus far.

## Introduction

Soft tissue sarcomas (STSs) are a rare and heterogenous group of mesenchymal cell tumors. The annual incidence is approximately 4 cases per 100,000 population cases per year in the United States and is more common in the pediatric population. More than half of STS cases are high-grade undifferentiated pleomorphic sarcomas, leiomyosarcomas, liposarcomas, synovial sarcomas, myxofibrosarcomas, or malignant peripheral nerve sheath tumors. The most common anatomical sites are the extremities. The rarity and heterogeneity of STS make them one of the most challenging tumors to manage.

## Case presentation

**Figure 1:**
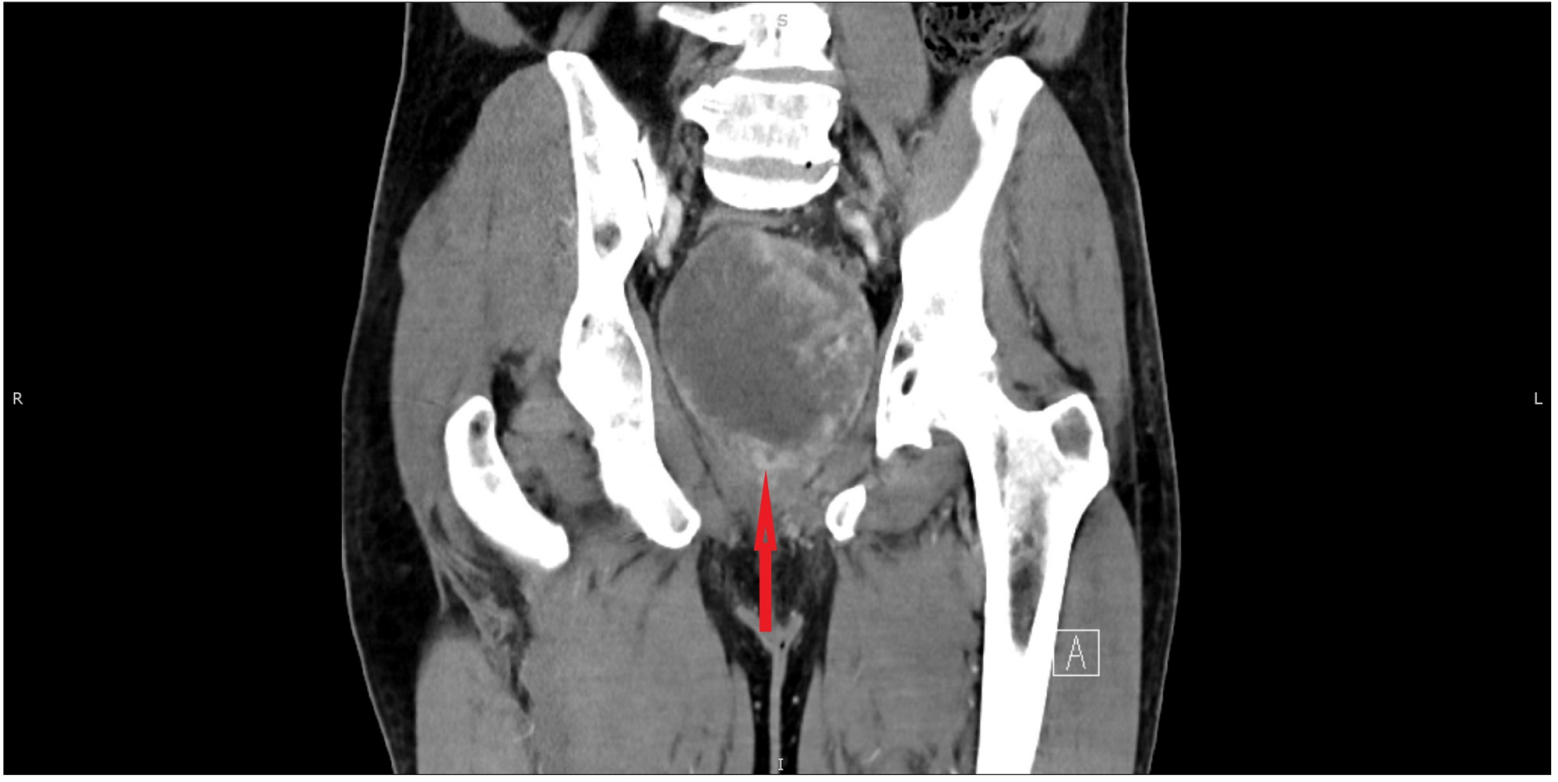
CT (coronal view) of the abdomen and pelvis with contrast showing large pelvic mass (red arrow)

**Figure 2:**
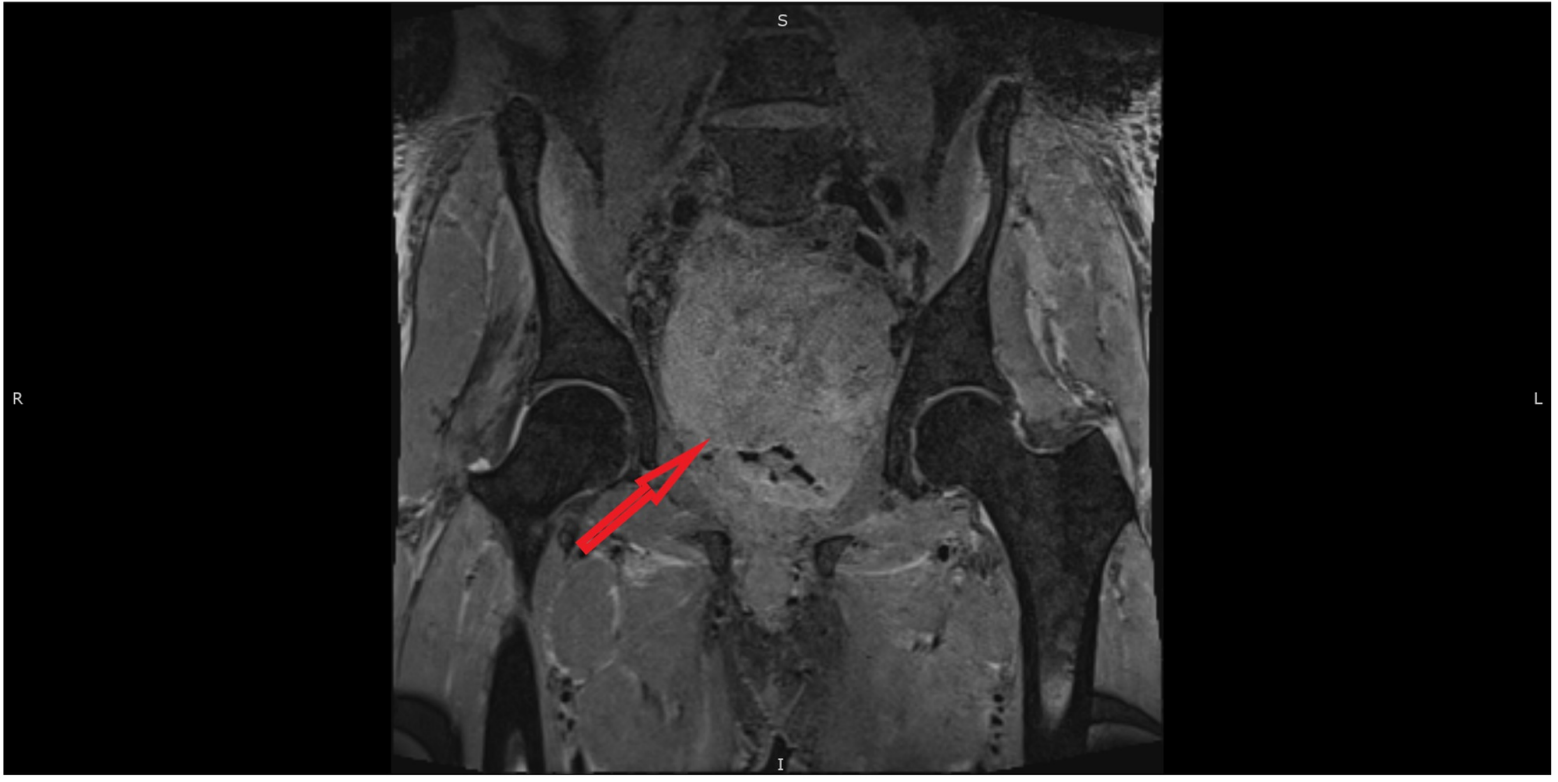
MRI (coronal view) of the pelvis showing large pelvic mass (red arrow)

**Figure 3:**
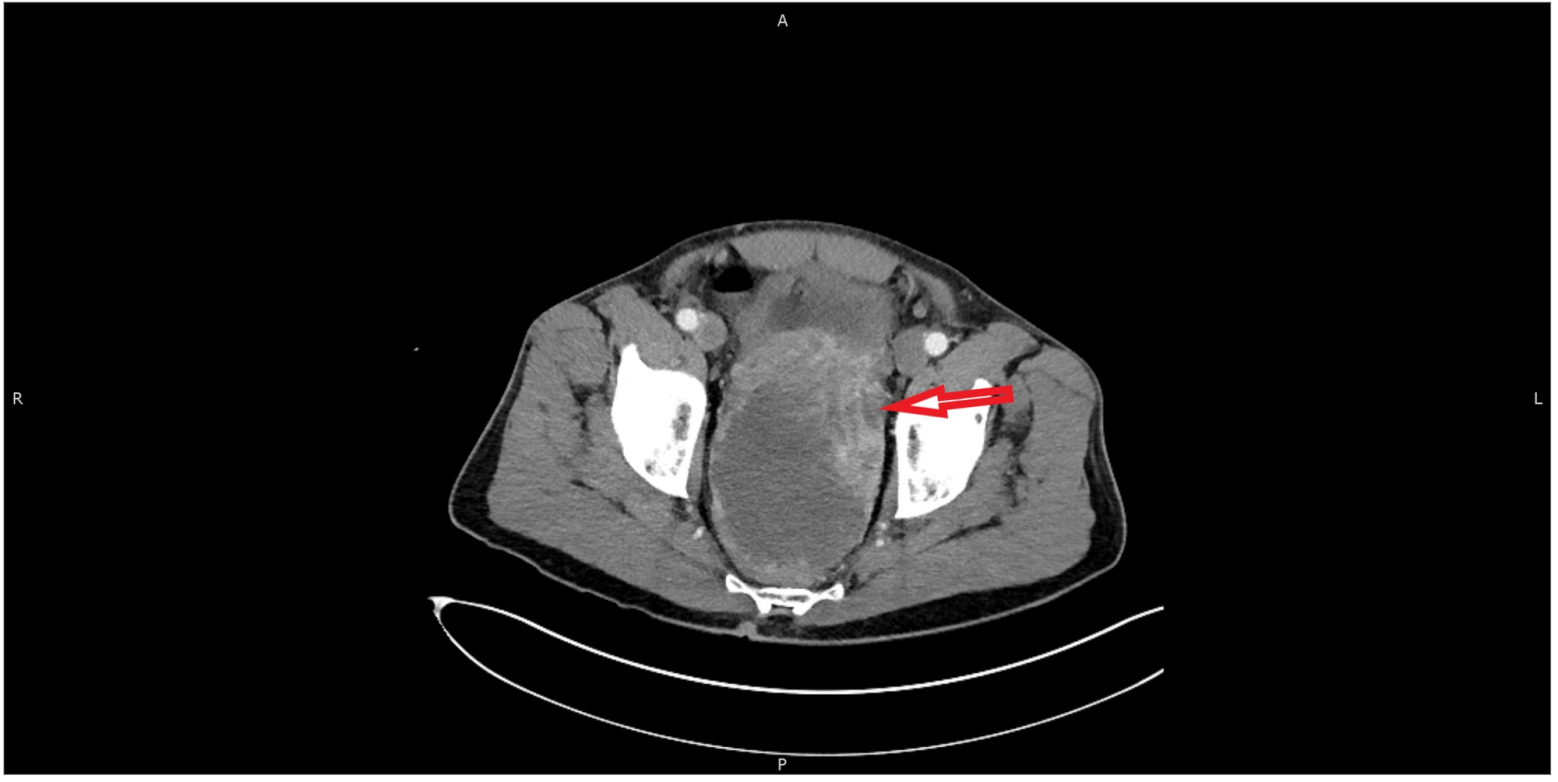
MRI (axial view) of the pelvis with contrast showing large pelvic mass fistulizing with the rectum (red arrow)

A 68-year-old male presented to the primary care physician with difficulty in passing urine for two days. His past medical history was significant for hypertension only. His symptoms were attributed to enlarged prostate gland, and he was sent home on tamsulosin. In the subsequent days, his symptoms worsened and he presented to the emergency department with severe lower abdominal pain, inability to void, and bloody diarrhea. Initial vitals were stable. Emergent bladder scan showed that approximately 800 mL of urine was retained in the bladder. Subsequently, straight urinary catheterization was performed, which retrieved 900 mL of urine, with remarkable improvement in his symptoms. Initial laboratory workup including blood urea nitrogen, serum creatinine, electrolytes, hemoglobin, white blood cell count, platelet count, and lactic acid were all within normal limits. Stool studies were unremarkable, and urine culture did not grow any microorganism. Stool cytology revealed numerous malignant spindle cells. Computed tomography (CT) of the abdomen and pelvis with contrast performed in the emergency department was remarkable for a 14.0 x 9.1 cm heterogenous solid mass fistulizing into the rectum (Figure 1), distension of the small bowel, and bilateral moderate hydronephrosis with possible hydroureter. A urinary catheter was placed, and broad-spectrum antibiotics including ciprofloxacin and metronidazole for bloody diarrhea was initiated, and he was admitted for further medical optimization. To better identify the mass and its extent for possible surgical debulking, magnetic resonance imaging (MRI) of the abdomen and pelvis with contrast was also performed, which was remarkable for large pelvic mass fistulizing with the rectum (Figures 2, 3). The differentials of the pelvic mass included lymphoma and STSs, and core needle biopsy was performed. The histopathology results showed malignant spindle and epithelioid neoplasm with necrosis consistent with high-grade sarcoma. Immunohistochemical stain (IHC) results were as follows:

1) Vimentin: positive.

2) Actin and caldesmon: weakly positive.

3) Paired box gene-8: rarely positive.

4) Mixed cytokeratin, AE1, AE3, desmin, S100, PSA, PSAP, CD34, CD117, myogenin, and tyrosinase: negative.

A multidisciplinary team including a musculoskeletal oncologist, urologist, radiation oncologist, and colorectal surgeon was formulated, and radiation therapy (RT) in an attempt to shrink the tumor was initiated. Finally, the patient underwent pelvic exenteration surgery considering extensive local tumor burden, and systemic chemotherapy was planned afterward. This is a very rare case of STS of the pelvis presenting with acute urinary retention and bloody diarrhea.

## Discussion

Sarcomas are a very rare and heterogenous group of human malignant tumors of mesenchymal cells. They comprise less than 1% of all adult malignancies but are relatively common in the pediatric population, with an estimated incidence of 4 per 100,000 cases per year [1]. Most of the sarcomas originate from soft tissues including skeletal and smooth muscles, adipose tissues, and fibrous tissue, and the rest originate from bone. Gastrointestinal stromal tumors are the most common sarcomas.

According to the World Health Organization, most of the soft tissue neoplasms are classified according to the presumptive tissue of origin such as liposarcoma, synovial sarcoma, leiomyosarcoma, rhabdomyosarcoma, fibrosarcoma, and angiosarcoma. In some cases where histogenesis is uncertain, sarcomas are designated based on the architectural pattern, e.g., alveolar sarcoma of soft parts, epithelioid sarcoma, clear cell sarcoma, and spindle cell sarcoma [2]. IHCs are often used as a diagnostic aid to differentiate between the origin of malignant cells, for example, chromogranin is specific for neuroectodermal tissue, myogenin for skeletal muscle, and cytokeratin for epithelium. IHC in our patient was positive for vimentin, weakly positive for actin and caldesmon, rarely positive for PAX-8, and negative for others. TNM staging is also widely used to classify STS and used as a guide for treatment in retroperitoneal/intra-abdominal sarcomas [3]. Our patient was staged as IIIB (T3N0M0).

STSs most commonly present with a slowly enlarging painless mass. It could also rarely present with symptoms of compressions, including extremity edema and sensory deficits. Our patient initially presented with urinary retention and later with bloody bowel movements, making presentation very rare in itself. Several anatomical sites are identified where STS could potentially occur, with a majority of cases found in the extremities [4]. Sarcomas are predominantly spread through the hematogenous route, but metastasis at the time of presentation is very uncommon and lungs are predominantly affected [5].

MRI is usually the initial imaging modality for most of the sarcomas, but some authors prefer CT for sarcomas confined to the retroperitoneum. In one study, Panicek et al. found no statistically significant difference between MRI and CT for the diagnosis of STS [6]. Positron emission tomography (PET) is not routinely recommended in the diagnosis of sarcomas as it cannot effectively differentiate between soft tissue tumors from low-to-intermediate sarcomas [7]. CT of the chest is recommended for the evaluation of pulmonary metastatic disease in all patients. Histopathological evaluation is required for diagnosis as well as treatment planning. Core needle biopsy is the preferred method of initial tissue sampling because of its high diagnostic accuracy [8]. Other options include fine needle aspiration and incisional biopsy.

The treatment of STSs is highly individualized and controversial. Several factors including tumor sizes, anatomic site, tumor grade, high-risk features, and range of histology must be considered before initiation of therapy. A multidisciplinary team including surgery, orthopedic surgery, medical oncology, and radiation oncology should be formulated [9]. In general, surgical excision alone is usually reserved for patients with low-grade, small (<5 cm), and superficial (superficial to the fascia) sarcomas. Patients with large, locally advanced, and unresectable STS are encouraged to participate in clinical trials. Neoadjuvant chemotherapy in an attempt to shrink the tumor for possible resection should be considered in patients who can tolerate systemic therapy [10]. The addition of RT to the surgical resection has a specific role in some of the inoperable extremity sarcomas and has shown to decrease local recurrence and amputations [11,12], without improvement in overall survival. Several benefits are proposed in the literature of preoperative RT in resectable STS including increase in R0 resection rates, precision of gross tumor volume, better radiation field with minimal adhesions from surgery, and possibility of resectibility [13]. Considering the extent of the tumor in our patient, preoperative RT was given to downsize the tumor and improve the chances of resectibility.

The heterogeneity and the rarity of STS have been obstacles to generating reliable evidence in favor of or against adjuvant and neoadjuvant chemotherapy. The 2014 European Society for Medical Oncology (ESMO) clinical practice guidelines state that adjuvant chemotherapy, though not the standard of care, could be considered as an option for STS greater than 5 cm and intermediate- to high-grade sarcomas [9]. Chemotherapy options include doxorubicin, epirubicin, and ifosfamide alone or in combination. Our patient underwent four cycles of RT followed by pelvic exenteration surgery and will be started on adjuvant chemoradiotherapy afterward. Lungs are the most commonly involved site in metastasis and are commonly managed with resection of the metastatic lesion [14].

Histological grade of the tumor at presentation, outcomes of local resection, and extent of distant metastasis are considered as major prognostic factors. Several nomograms including the Memorial Sloan Kettering Cancer Center sarcoma nomogram and Helsinki University sarcoma nomogram have been developed to predict post-operative survival more accurately [15,16]. In most of these nomograms, histological grade was of major significance [17].

## Conclusions

STSs are a rare but very diverse group of tumors of mesenchymal cell origin. The complexity and heterogeneity of these tumors have been the major obstacles in their effective treatment. Several treatment options including local resection, RT, and adjuvant chemotherapy have been studied, but the standard care is still not defined. STS of the pelvis is a rare entity, which can present with significant anatomic complications such as urinary retention and fistula, as evident in our patient. Early diagnosis and prompt treatment with debulking surgery with and without radiation is crucial in the management of these tumors.
